# Complete genome sequence of *Anaerocolumna* sp. MB42-C2 isolated from a hot spring in Qingliu, Fujian, China

**DOI:** 10.1128/mra.00711-26

**Published:** 2026-09-21

**Authors:** Jingjing Zhao, Yichia Lin, Chih-Hung Wu, Sheng-Chung Chen, Wanling Qiu, Guowen Dong, Chao-Jen Shih, Yen-Chi Wu, Wangchung Xiao

**Affiliations:** 1 School of Resources and Chemical Engineering, Sanming University 66283, Sanming City, Fujian, People’s Republic of China; 2 Fujian Provincial Key Laboratory of Resources and Environmental Monitoring and Sustainable Management and Utilization, Sanming University 66283, Sanming City, Fujian, People’s Republic of China; 3 School of Entrepreneurship Management, Sanming University 66283, Sanming City, Fujian, People’s Republic of China; 4 College of Environment and Safety Engineering, Fuzhou University 12423, Fuzhou City, Fujian, People’s Republic of China; 5 Bioresource Collection and Research Center, Food Industry Research and Development Institute 63418, Hsinchu City, Taiwan, People’s Republic of China; California State University San Marcos, San Marcos, California, USA

**Keywords:** *Anaerocolumna*, anaerobes, hot spring

## Abstract

Here, we report the complete genome sequence of *Anaerocolumna* sp. MB42-C2 isolated from a hot spring in Qingliu, Fujian, China. The genome comprises 6,437,239 bp with a G + C content of 36.49 mol% and provides a genomic basis for further species delineation and characterization of genomic features.

## ANNOUNCEMENT

Strain MB42-C2 was isolated from a hot spring water sample collected from Tianfang Yuetan hot spring, Qingliu, Fujian, China (26.1762°N, 116.9392°E) on 3 July 2021. Hot spring water was collected under ambient conditions in a sterile bottle and subsequently inoculated into anaerobic modified DSM 924 medium, which was incubated at 25°C for 2 weeks. The medium contained, per liter, 1 g MgCl_2_·7H_2_O, 0.5 g KCl, 0.1 g CaCl_2_·2H_2_O, 0.4 g K_2_HPO_4_, 1 g NH_4_Cl, 10 mL trace element solution, 2 g yeast extract, 2 g tryptone, 0.5 mL Na-resazurin solution (0.1%), 4 g NaHCO_3_, 10 mL vitamin solution, 0.25 g L-cysteine-HCl·H_2_O, and 0.25 g Na_2_S·9H_2_O, with an N_2_/CO_2_ (4:1) headspace. The enrichment culture was serially diluted in anoxic medium, mixed with molten agar, and rolled horizontally to form a thin agar layer, a procedure known as the roll-tube technique (1). Individual colonies were picked and repeatedly purified using the same procedure at 25°C. The isolate was identified by 16S rRNA gene clone sequencing using primers 8F and 1492RU (2), and culture purity was confirmed by morphological observation, 16S rRNA gene analysis, and genome sequencing. Whole-genome sequencing was performed to clarify the taxonomic placement of strain MB42-C2 and enable characterization of its genomic features.

Strain MB42-C2 has been deposited in the Bioresource Collection and Research Center, Taiwan, as BCRC 81438. For genome sequencing, the strain was cultivated in modified DSM 924 medium at 30°C, and genomic DNA was extracted using the NucleoBond HMW DNA Kit (Macherey-Nagel, Germany) according to the manufacturer’s instructions. Genomic DNA extraction yielded approximately 6 μg of DNA. Genome sequencing was performed by Sangon Biotech (Shanghai) Co., Ltd. using the DNBSEQ-T7 platform (MGI Tech Co., Ltd.) and the MinION sequencer (Oxford Nanopore Technology).

For DNBSEQ-T7 sequencing, 1 μg genomic DNA was sheared to approximately 300 bp and used to prepare a paired-end library with the Hieff NGS MaxUp II DNA Library Prep Kit. The library was sequenced using the DNBSEQ-T7 platform in PE150 format, generating 9,756,898 reads (Table 1). Raw reads were processed using Trimmomatic v0.36 (3) to remove ambiguous bases and adapter sequences, trim low-quality bases (Q < 20) from both ends using a 5 bp sliding window, and discard reads shorter than 35 nt together with their paired reads.

**TABLE 1:** Genome characteristics of *Anaerocolumna* sp. MB42-C2

Characteristic	Value
Sequencing and assembly features	
No. of MGI-DNBSEQ-T7 total reads	9,756,898
No. of Nanopore total reads	137,688
Total no. of MGI-DNBSEQ-T7 bases	1,463,534,700 bp 6,381 bp
Total no. of Nanopore bases	835,957,064 bp
Genome coverage (MGI; Nanopore)	227×; 130×
Genome features	
Chromosome size (GC content)	6,437,239 bp (36.49%)
Total no. of genes	5,707
No. of coding sequences	5,620
No. of pseudogenes	61
No. of rRNAs	21
No. of tRNAs	61
No. of noncoding RNAs	5

**Fig 1:**
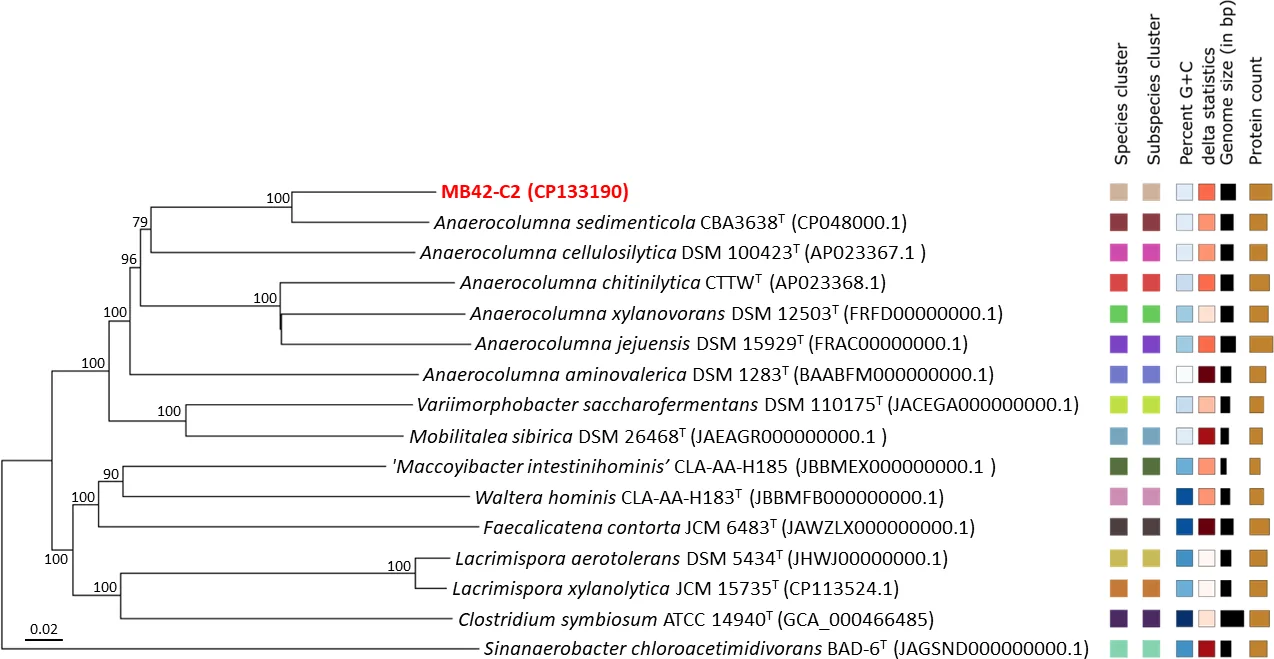
Whole genome-based phylogenetic tree of strain MB42-C2 and related type strains generated using the Type (Strain) Genome Server. The tree was inferred with FastME 2.1.6.1 from Genome BLAST Distance Phylogeny distances calculated from genome sequences. Branch lengths were scaled according to GBDP distance formula d5. Blue numbers at the nodes indicate GBDP pseudo-bootstrap support values greater than 60% based on 100 replications, and red numbers indicate branch lengths. The tree was midpoint-rooted. The colored squares indicate species clusters, subspecies clusters, genomic G + C contents, *δ* statistics, genome sizes, and protein counts.

For MinION sequencing, 1 µg of unsheared genomic DNA was used to prepare a ligation library with the SQK-LSK109 kit. The library was sequenced on an R9.4.1 flow cell. Reads were base-called using Guppy v6.5.7 in high-accuracy mode, trimmed with Porechop v0.2.4 (4), and filtered with NanoFilt v2.8.0 (5), resulting in 137,688 reads with an average length of 6,071 bp, an *N*_50_ of 11,383 bp, and a total yield of 835,957,064 bp (Table 1). Long reads were assembled using Canu v2.2 (6), and the resulting assembly was polished with DNBSEQ-T7 short reads using Pilon v1.18 (7). Overlapping ends were identified and trimmed to generate a circular chromosome, and no genome rotation was performed. The average short- and long-read coverages were 227× and 130×, respectively. Gene prediction and annotation were performed using National Center for Biotechnology Information (NCBI) Prokaryotic Genome Annotation Pipeline (PGAP) v6.6 (8). The Type (Strain) Genome Server (TYGS) whole-genome analysis (9) placed strain MB42-C2 as a sister lineage to *Anaerocolumna sedimenticola* CBA3638ᵀ (10) with 100% pseudo-bootstrap support while assigning the two strains to separate species clusters (Fig. 1).

The final assembly consisted of a single circular chromosome of 6,437,239 bp with a G + C content of 36.49%, containing 21 rRNA and 61 tRNA genes (Table 1). Default parameters were used unless otherwise specified.

## Data Availability

The genome sequence of strain MB42-C2 has been deposited in GenBank under accession number CP133190. The version of the genome described in this paper is the first version. The BioProject and BioSample accession numbers are PRJNA1006852 and SAMN37052272, respectively. MinION and DNBSEQ-T7 raw reads were deposited in the Sequence Read Archive under accession numbers SRR27218776 and SRR27218777, respectively.
